# Influence of Polymer Concentration on the Viscous and (Linear and Non-Linear) Viscoelastic Properties of Hydrolyzed Polyacrylamide Systems in Bulk Shear Field and Porous Media

**DOI:** 10.3390/polym16182617

**Published:** 2024-09-15

**Authors:** Madhar Sahib Azad

**Affiliations:** Department of Petroleum Engineering, College of Petroleum and Geoscience, King Fahd University of Petroleum and Minerals (KFUPM), Dhahran 31261, Saudi Arabia; madhar.azad@kfupm.edu.sa

**Keywords:** shear rheometry, porous media rheometry, polymer concentration effect, viscosity, linear viscoelasticity, non-linear viscoelasticity, shear thickening

## Abstract

Enhanced oil recovery (EOR) methods are generally employed in depleted reservoirs to increase the recovery factor beyond that of water flooding. Polymer flooding is one of the major EOR methods. EOR polymer solutions (especially the synthetic ones characterized by flexible chains) that flow through porous media are not only subjected to shearing forces but also extensional deformation, and therefore, they exhibit not only Newtonian and shear thinning behavior but also shear thickening behavior at a certain porous media shear rate/velocity. Shear rheometry has been widely used to characterize the rheological properties of EOR polymer systems. This paper aims to investigate the effect of the polymers’ concentrations, ranging from 25 ppm to 2500 ppm, on the viscous, linear, and non-linear viscoelastic properties of hydrolyzed polyacrylamide (HPAM) in shear field and porous media. The results observed indicate that viscous properties such as Newtonian viscosity increase monotonically with the increase in concentration in both fields. However, linear viscoelastic properties, such as shear characteristic time, were absent for concentrations not critical in both shear rheometry and porous media. Beyond the critical association concentration (CAC), the modified shear thinning index decreases in terms of concentration in both fields, signifying their intensified thinning. At those concentrations higher than CAC, the viscoelastic onset rate remains constant in both fields. In both fields, the shear thickening index, a strict non-linear viscoelastic property, initially increases with concentration and then decreases with concentration, signifying that the polymer chains do not stretch significantly at higher concentrations. Also, another general observation is that the rheological properties of the polymer solutions in both porous media and shear rheometry only follow a similar trend if the concentration is higher than the CAC. At concentrations less than the CAC, the shear and porous media onset rates follow different trends, possibly due to the higher inertial effect in the rheometer.

## 1. Introduction

The oil industry can be classified into upstream, midstream, and downstream. The upstream oil industry involves exploration, drilling, reservoir management, and production operations. Reservoir engineering is the application of scientific and engineering principles to production from the developed reservoir for a maximum economic return [1]. If the reservoir of interest is characterized by higher oil viscosity and the thermal EOR methods are deemed appropriate as per EOR screening criteria, these methods may be applied [2,3]. Reservoir engineers must be familiar with thermodynamics—a scientific concept. In addition, by optimizing the recovery factor, reservoir engineers are also responsible for determining the volume of the oil and gas in situ, understanding the primary drive mechanisms, determining the various reservoir properties using pressure transient analysis, etc. [4,5,6].

Oil recovery, in general, can be classified into three stages—primary, secondary, and tertiary. Reservoir engineers are responsible for finding the reasons behind poor recovery factors during primary and secondary drives, and they must propose and design appropriate enhanced oil recovery (EOR) methods to improve recovery factors beyond secondary water floods. Poor recovery factors observed during water floods could be attributed to a water flood’s poor sweep and displacement efficiency. Water’s low viscous nature and poor interfacial activity mean it does not provide sufficient mobility control or mobilization. Although EOR methods are generally categorized based on the injectant nature as thermal, miscible, or chemical methods, they can also be categorized into mobilization and mobility control methods based on the operational mechanisms [7,8]. The higher the mobility of the injection fluids, the easier the opportunity for intense fingering through heavy oil. Mobility control methods ensure that the mobility of the injection fluid is not adversely high. Water flooding is a poor mobility control method as the viscosity of the water tends to be on the lower side. The mobility of the injection fluid can be controlled by increasing the viscosity of the injection fluids. Polymer flooding is a prime example, where the addition of polymers to the injection water increases the viscosity of the injection fluids and reduces its mobility to ensure a good sweep efficiency. However, there is a limit for oil viscosity up to which a polymer flood can efficiently displace the heavy oil [9]. Nevertheless, polymer flooding is one of the most widely applied matured EOR methods applicable for reservoirs ranging in oil viscosity from 10 cP to 3000 cP [9,10,11,12,13,14,15,16,17]. The polymer concentration must be designed to achieve a favorable mobility ratio depending on the oil viscosity. Unlike previous polymer flooding practices, most recent polymer flood projects employ high concentrations and larger sizes [14]. Concentration is one of the most critical design parameters for light and heavy oil polymer flood projects. A careful look at Table 3 in a study by Sagyndikov et al. [18] reveals that the average incremental recovery for polymer flood projects conducted across the globe ranges between ~3 and 18%. The varying range could be due to differences in permeability, oil viscosity, operational parameters, the injected polymer solution’s properties, etc. In general, the higher the oil viscosity, the lower the incremental recovery factor if the permeability of the rocks is similar. This can be understood by looking at Figure 4 in Asghari and Nakutnyy’s study [19], where polymer injections of varying concentrations, ranging from 500 ppm to 10,000 ppm, into an unconsolidated core saturated with 1000 cP oil lead to an over 20% greater recovery than in those cases where similar concentrations of polymer were injected into an unconsolidated core saturated with 8000 cP oil. 

Xanthan gum and hydrolyzed polyacrylamide (HPAM) are the most widely used EOR polymers [20,21]. Other polymer-based systems, such as associative polymers [22], viscoelastic surfactants [23], and ter-polymers [24], have also been evaluated for EOR applications. Xanthan gum is characterized by rigid chains and does not stretch. HPAM is cheaper and a synthetic polymer characterized by flexible chains. It has been reported that polymer solutions that thin in the shear field thicken in the extensional field [25]. The extensional field is a shear-free field with no shearing effect, whereas the bulk shear field has no extensional deformation. Flexibility in HPAM chains induces viscoelastic effects. Viscoelasticity in the polymer solutions can lead to a high pressure drop, reduced injectivity [26,27,28] in unfractured conditions, and higher oil recovery at favorable conditions [29,30,31,32,33,34,35]. Shear thickening viscoelastic effects occur when polymer solutions characterized by higher elasticity (stretchability) do not have sufficient time to relax. In other words, if the observation time is too short relative to the relaxation time of the polymer solutions, then the viscoelastic effect would be manifested as an increase in resistance concerning the rise in the rate. Please note that the shear rate is inversely proportional to observation time. This leads to strain hardening in the extensional field [36], porous media thickening in porous media [37], and shear thickening in the bulk shear field [37,38]. A closer look into the onset rate for all these fields indicates that if the field is strong, as in the extensional field, shear thickening can happen at an even lower rate [36,38]. While the shear thickening of EOR polymer systems in extensional and porous media is very well-documented [26,27,28,34,35,39,40], it was only in our recent paper that shear thickening in the bulk shear field was interpreted and analyzed in detail [37]. Azad [37] demonstrated that HPAM, conventionally believed to be shear thinning in the shear field, will also thicken in the shear field if it has sufficient viscoelasticity. The author investigated the individual effect of salinity and the combined effect of concentration, Mw, on the shear thickening and correlated it with the effect of pressure drop/resistance factor obtained in each case. The author also used viscous xanthan gum and viscoelastic HPAM and analyzed the thickening intensity to affirm the role of the polymer’s viscoelasticity on shear thickening [37]. It is worth highlighting that Howe et al. [41] also observed the shear thickening with EOR-HPAMs in the shear field. Several non-EOR studies have lighted the utility of high-speed shear flow in inducing strong flow [42,43]. Further, while linear viscoelasticity has been conventionally used to correlate the polymer’s viscoelastic behavior in porous media, several recent pieces of literature emphasize the relevance of non-linear viscoelasticity over linear viscoelasticity for porous media applications [21,35,36,37,41,44].

In general, the higher the salinity, the higher the non-linear viscoelastic effects in all fields—bulk shear [37], bulk extensional [34,35], and porous media [45,46]. It is also important to highlight that the high salinity mentioned referred [34,35,45,46] does not have same concentration. The lower the permeability, the higher the non-linear viscoelastic effects [47]. The higher the Mw, the stronger the thickening in the bulk extensional field [34], bulk shear [37], and in the porous media [21]. It is essential to highlight that the high Mw mentioned in the previous sentence comes with the lowest concentration and vice versa. However, the polymer concentration by itself is a tricky parameter, and it depends on the regime in which the polymer concentration is. At concentrations less than the critical concentration, the polymer chains tend to be in the extended state, and Clasen [48], in their classical paper “How dilute are dilute solutions extensional flows?” found the macromolecular chains are in a stretched state which eventually leads to having a higher extensional relaxation time than oscillatory relaxation time. Due to the nature of the extensional flow, the polymer chains were in the stretched state when their concentration was less than the overlap concentration. On the other hand, polymer chains become entangled at higher concentrations, and they do not stretch significantly. An increase in concentration may not result in increased non-viscoelastic effects. Non-linear viscoelastic effects are more relevant in porous media but poorly understood. Azad [37] studied the non-linear viscoelastic effects of polymer systems using steady-shear rheometry at varying salinity and the combined impact of concentration–Mw. In this work, the effect of concentration on HPAM is studied in the bulk field and compared with the porous media rheogram reported in the literature [45]. Salinity, temperature, and Mw are all kept the same to analyze how the polymer concentration influences the shear-based viscous, linear–viscoelastic, and non-linear viscoelastic behavior of HPAM in both shear field and porous media. The properties to be studied are the Newtonian viscosity, shear characteristic time, shear thinning index, viscoelastic onset rate, and shear thickening index. It is worth highlighting that Jouenne and Levache [49] have developed a comprehensive model for predicting the non-Newtonian behavior of EOR polymer systems characterized by various concentrations, Mw, salinity, and temperature. However, the authors did not study the non-linear viscoelastic properties (such as shear thickening), and studying and comparing the effect of concentration on the non-linear viscoelastic properties in both shear field and porous media properties is one of the novelties of this work. 

## 2. Experimental

The materials and polymer system formulations are as follows: HPAM is the commonly used EOR polymer. HPAM 3630 with a Mw of 18–20 MDa is used in this work. The salinity is fixed to 2.52% TDS with the 2.3% NaCl + 0.22% NaHCO_3_. SNF (Andrézieux-Bouthéon, France) supplied HPAM, and Lab-Chem supplied the salts. Initially, 2.52% TDS brine was prepared by adding 2.3% NaCl and 0.22% NaHCO_3_ salts into distilled water. Once the saline brine was prepared, the polymer solutions of appropriate concentrations were prepared by dissolving the polymer powder in brine at a low rotational speed of 200 rev/min for ~24 h. In total, eight different HPAM 3630 systems with varying concentrations were considered. It is important to reiterate that all the variables, such as the salinity, Mw, polymer nature, and temperature, are constant except for the polymer’s concentrations. For this salinity and similar Mw (20 MDa), 200 ppm has been reported to be the overlap concentration [45]. Therefore, the chosen concentrations are 25 ppm, 50 ppm, 100 ppm, 480 ppm, 900 ppm, 1600 ppm, and 2500 ppm, and the preferred ranges include those below and above the overlap concentration. The rationale for selecting these ranges is that it is essential to understand how the viscous, linear viscoelastic, and non-linear viscoelastic properties would be influenced in steady-shear and porous media when the concentration range is below and above the overlap concentration. 

Rheometer: A rotational rheometer capable of measuring shear rheology, purchased from Anton Paar (Graz, Austria), was used. The equipment model is MCR 702. Concentric cylinder (CC) geometry is used. The bob length is 40 mm, and the cup diameter is 28.91 mm. The gap distance is zero. It is essential to highlight that the lower the gap distance, the higher the elastic effects [50]. A careful examination of Figure 10a of Howe et al. [41] revealed that a 4° cone angle results in the early onset of shear thickening of HPAM polymer systems than a 1° cone angle, suggesting that the higher cone angle amplifies the elastic effects. Shear rheometry equipped with CC geometry can induce shear thickening of the polymer system unless characterized by a low Mw, high concentration, or highly concentrated rigid structures [37]. Recently, Hamad et al. [51] reported that employing a non-circular geometry would enable the shear thickening for even highly viscous polymer systems such as those characterized by low Mw-high concentration. We employed the commonly used CC geometry to describe the rheological properties of high-Mw flexible HPAM polymers. The steady-shear rheological experiments are performed from the shear rate of 0.01 s^−1^ to 1000 s^−1^. All the experiments were performed at room temperature so that the effect of concentration on the rheological behavior could be studied without any additional influence due to temperature. Each experiment was repeated at least twice. 

The polymer systems’ preparation was as follows: Polymer solutions were prepared by dissolving the polymer powder at the concentration of interest by low-speed rotation for around 24 h. The brine of 2.52% TDS (total dissolved salt) was prepared by adding 2.3% of NaCl and then 0.22% of NaHCO_3_ in distilled water. 

## 3. Results and Discussion

**Figure 1:**
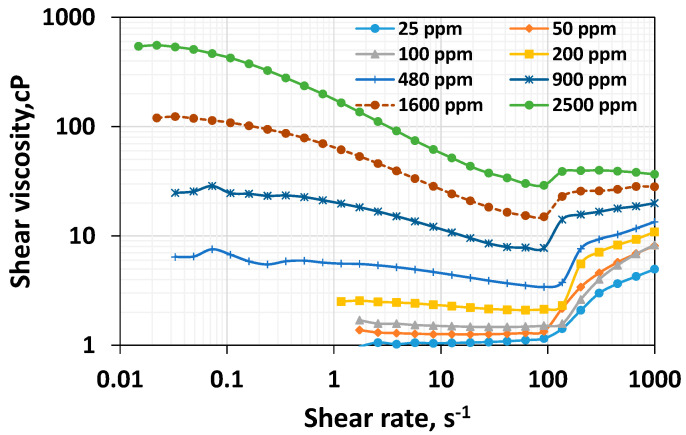
Effect of polymer concentration on the shear rheological behavior of HPAM systems.

The typical flow regime for the non-Newtonian polymer solutions in steady-shear rheometry is Newtonian and shear thinning. However, Azad [37] recently demonstrated that while a viscoelastic polymer system can provide a shear-thickening at high-shear rates in shear rheometry, the viscous polymer system shows a continuous shear thinning at low shear rates. This work also offers rheograms for Newtonian, shear thinning, and shear thickening regimes for the polymer systems of varying concentrations (Figure 1). The Newtonian viscosity, shear characteristic time, modified shear thinning index, viscoelastic onset rate, and altered shear thickening index for all the studied systems are provided in Table 1. The raw shear rheological data for all the studied concentrations are shown in Table 2. 

### 3.1. Effect of Concentration on Newtonian Viscosity of Polymer System

The shear viscosity as a function of shear rate for polymer systems of varying concentrations is shown in Figure 1.

**Figure 2:**
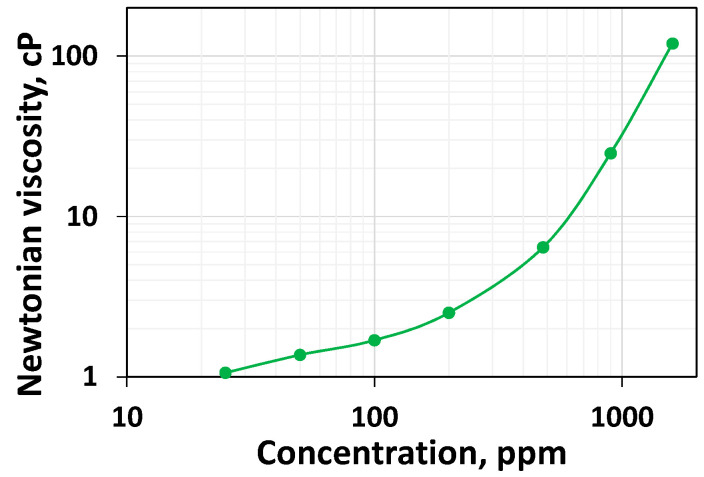
Newtonian viscosity as a function of polymer concentration for HPAM systems.

As expected, the higher the concentration, the higher the viscosity throughout the range of shear rates. However, a rheologically more exciting phenomenon can be interpreted. Newtonian viscosity is the relatively constant viscosity that would last until the shear rate becomes high enough to induce shear thinning. It is essential to highlight that for the lower concentration, the viscosity values at low shear rates are unstable; hence, they are omitted. Although it is clear that the increase in concentration leads to a rise in Newtonian viscosity, the rate of increase is higher at higher concentrations. Figure 2, which plots the Newtonian viscosity as a function of concentration, shows this trend clearly. The viscosity values for 25 ppm, 50 ppm, and 100 ppm polymer systems are 1.06 cP, 1.37 cP, and 1.69 cP, respectively. This indicates they are increasing moderately. For 200 ppm, 480 ppm, 960 ppm, 1600 ppm, and 2500 ppm, these values are 2.51 cP, 6.42 cP, 24.8 cP, 120 cP, and 542 cP, respectively. From 200 ppm, the increase in viscosity is steeper and it appears to be the overlap concentration, which is consistent with the value reported for the HPAM 3830 systems under similar conditions [45]. Above the critical concentration, the polymer systems are entangled and display a higher resistance to flow. The higher concentration of HPAM systems leading to higher viscosity has been reported in the literature [19]. The higher viscosity generally could lead to higher mobile viscous oil recovery in unconsolidated high permeable media [19]. However, the higher viscosity could not lead to higher residual heavy oil recovery [52]. Still, it could cause an injection pressure increase, which may reduce the injectivity in unfractured vertical well conditions or increase the chance of fracturing in the reservoir [53]. Further, the higher concentration of polymer would necessitate a relatively higher expenditure.

### 3.2. Effect of Concentration on the Shear Characteristic Time

**Figure 3:**
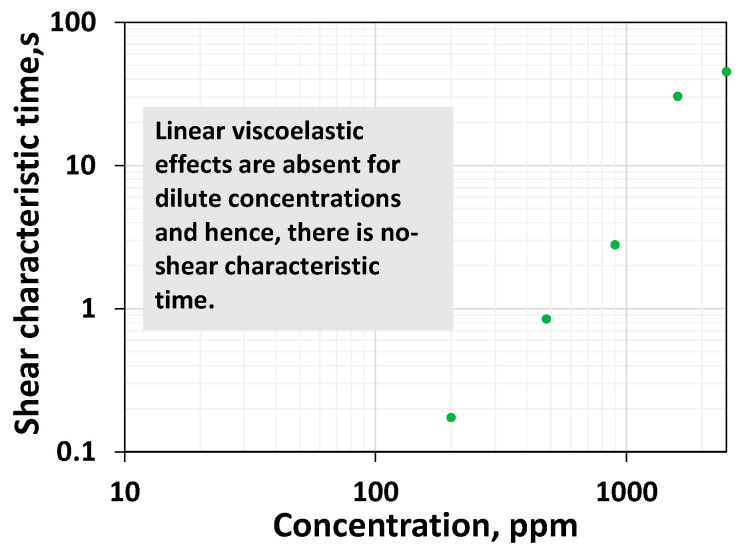
Shear characteristic time as a function of polymer concentration for HPAM systems.

The shear characteristic time is the time that corresponds to the polymer’s linear relaxation properties [54]. It is the inverse of the shear rate at which the transition occurs from the Newtonian to the shear thinning regime. For those concentrations less than 200 ppm, it is clear from Figure 1 that only the Newtonian regime prevails before the final thickening. No detectable shear thinning is evident for the systems with polymer concentrations of 25 ppm, 50 ppm, and 100 ppm. Since there is no shear thinning, it appears they have a negligible linear relaxation ability, highlighted by the zero value of the shear characteristic time. Figure 3 plots the shear characteristic time as a function of the polymer’s concentration, which indicates the absence of linear relaxation time within a dilute regime (low concentrations). From 200 ppm to 1600 ppm, the shear characteristic time increases substantially.

It can be said that an increase in the concentration above the overlap threshold increases the shear characteristic time, which is proportional to Newtonian viscosity.

### 3.3. Effect of Concentration on Shear Thinning

**Figure 4:**
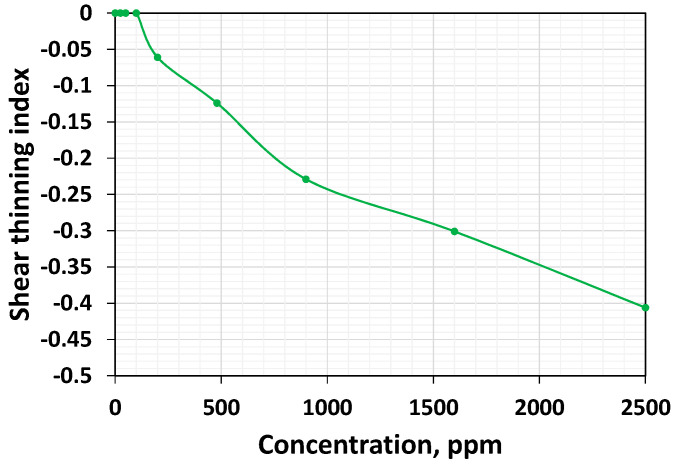
Shear thinning index as a function of concentration.

Shear thinning is an essential property for EOR applications. If the polymer tends to thin more, it is an indication that it can generate a higher resistance factor in the farthest portion of the reservoir as long as the species responsible for viscosity augmentation is not affected by mechanical degradation. The higher thinning index also indicates that if the flow field is dominated by shear as in the case of pipelines, or intermediate fluxes in the high-permeable media, they tend to lose viscosity. The effect of concentration on the shear thinning characteristic of the polymer system is studied by plotting the data points that fall between the rates that correspond to the transition from the Newtonian to the shear thinning regime and from the shear thinning to the shear thickening regime. The shear thinning index (calculated using the power-law fit) as a function of concentration is shown in Figure 4.

The shear thinning index for these systems is −0.061, −0.124, −0.229, −0.331, and −0.406, respectively. Above the critical concentration, the increase in the concentration leads to a monotonically increase in the magnitude of the shear thinning index (Figure 4), indicating a higher shear thinning intensity. As the concentration increases, more and more polymer chains are in an entangled and coiled state at zero shear rate/stress, and they can be unfolded and oriented on the molecular scale to the applying shearing force direction so that more significant polymer concentrations may lead to a higher thinning ability. 

### 3.4. Effect of Concentration on Viscoelastic Onset Rate

**Figure 5:**
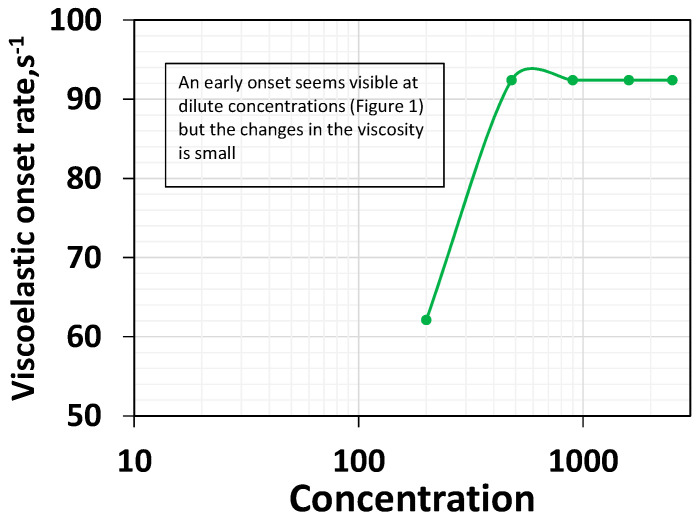
Viscoelastic onset rate as a function of polymer concentration.

The viscoelastic onset rate is the rate at which the polymer solutions begin showing shear thickening behavior. Shear thickening occurs when the polymer’s coils do not have sufficient time to relax. Generally, the higher the non-linear viscoelasticity, the lower the viscoelastic onset rate [37,41]. Figure 1 shows that shear thickening occurs with all the polymer systems. This is consistent with the porous media thickening behavior reported for HPAM 3830 systems under similar conditions [45]. This conveys that the HPAM polymer solutions exhibit viscoelastic characteristics at varying concentrations. The effect of polymer concentration on the viscoelastic onset rate is shown in Figure 5. 

The viscoelastic onset remains relatively constant concerning increasing concentration after 200 ppm. However, a careful look into Figure 1 indicates that an early onset rate was visible at a dilute low concentration. However, the increase in viscosity is marginal. Nevertheless, for concentrations above 200 ppm, the onset rate of 92.4 s^−1^ is constant (Figure 1 and Figure 5), consistent with Howe et al.’s observation [41]. Increasing a concentration above the critical concentration does not increase the non-linear viscoelastic property, such as the rouse relaxation time [41] or viscoelastic onset rate (Figure 5). Still, it will increase the linear relaxation time (Figure 3). In other words, an increase in the concentration above the critical concentration indeed leads to a rise in the linear viscoelastic property (Figure 3) but not the non-linear viscoelastic property (Figure 5) [41]. 

### 3.5. Effect of Concentration on Shear Thickening Index

**Figure 6:**
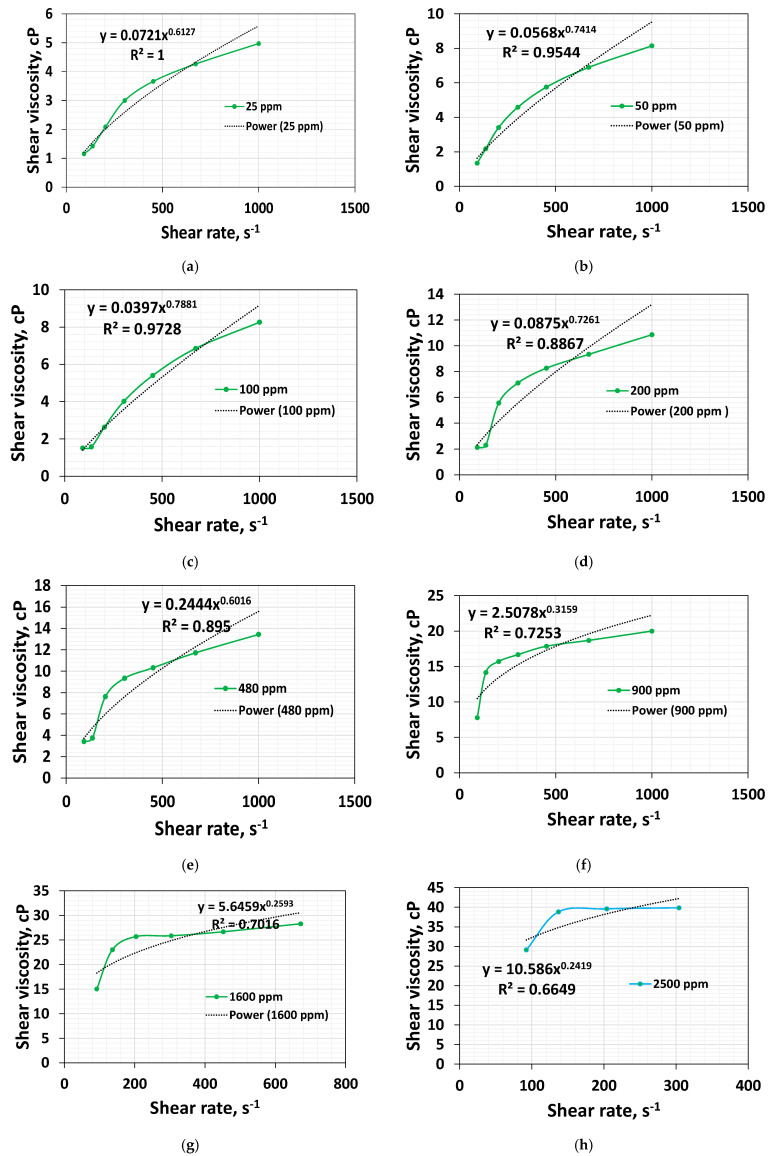
Power-law fit between two points extracted between the onset rate and 1000 s^−1^ for HPAM 3630 systems at (**a**) 25 ppm, (**b**) 50 ppm, (**c**) 100 ppm, (**d**) 200 ppm, (**e**) 480 ppm, (**f**) 900 ppm, (**g**) 1600 ppm, (**h**) 2500 ppm.

The shear thickening index quantifies the degree of the increase in viscosity for the rise in the shear rate. It can be obtained by fitting the power law (Oswald de Waele) to the shear thickening regime using Excel. The power law can model shear thinning and shear thickening [35,37]. Figure 6a–h shows the power-law fit to the apparent viscosity data in the shear thickening regime—one corresponding to the 92.4 s^−1^ and another corresponding to the rate at which maximum viscosity is observed. Please note that the onset rate is a little low at dilute concentrations. However, the viscosity increase is very marginal. We consider taking the first data points at 92.4 s^−1^ for all the concentrations. 

**Figure 7:**
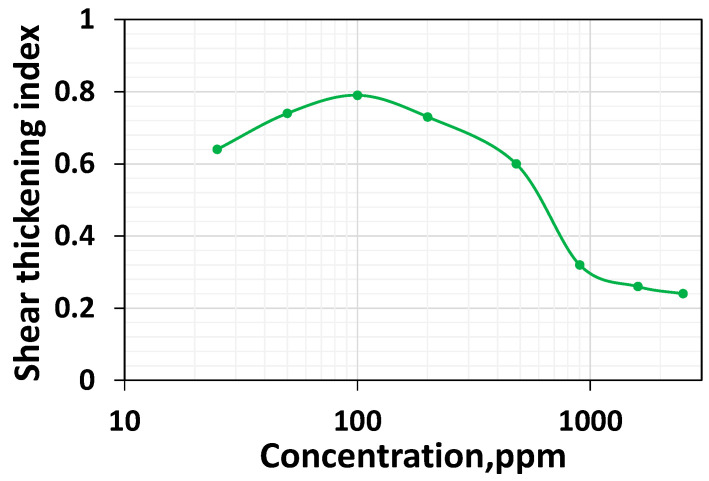
Shear thickening index (extracted from Figure 6a–h) as a function of the concentration of the investigated HPAM solutions.

The shear thickening index initially increases and decreases steadily after 200 ppm (Figure 7). 

This is because the polymer chain tends to be entangled and cannot stretch more due to the high-speed rotation when its concentration is higher. The decrease in shear thickening index with increased concentration at higher concentrations indicates that non-linear viscoelasticity and stretching ability are decreasing. It is worthwhile to highlight that Al-Hamad et al. [51] employed CC geometry in shear rheometry to characterize the rheology of HPAM 6030 polymer systems at the concentrations of 650 ppm, 1650 ppm, and 2350 ppm, respectively. With CC geometry, these systems’ shear thickening onset rates are 62.1 s^−1^, 62.1 s^−1,^ and 92.4 s^−1,^ respectively. However, while employing non-circular geometry, the authors reported the onset values of 12.7 s^−1^, 41.8 s^−1^, and 62.1 s^−1^, respectively, signifying that (a) at higher concentrations, the stretching ability decreases with increasing concentration; and (b) non-circular geometry can characterize the stretching ability of the polymer systems of varying concentrations more precisely. 

The results obtained using the shear thickening index in this work, along with the analysis of Al-Hamad et al. [51] using both methods of the shear thickening index, obtained using CC geometry, and the viscoelastic onset rate obtained using non-circular geometry could reveal the fact at higher concentrations, the polymer’s stretchability and non-linear viscoelasticity is reduced. 

### 3.6. Comparative Discussion between Various Rheological Parameters

**Figure 8:**
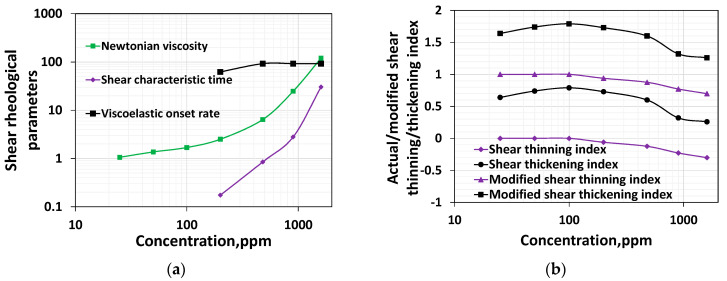
(**a**) Effect of concentration on all bulk shear rheological parameters; (**b**) Effect of concentration on the modified shear thinning and shear thickening index.

The effect of concentration on various properties, such as the Newtonian viscosity, shear characteristic time, and shear thickening index, is graphically displayed in Figure 8a. The shear thinning index cannot be shown in the log-log plot (Figure 8a) as they have zero and negative values. If the magnitude of the shear thinning indices is plotted without the minus sign, it would have a similar trend to the shear thickening index, i.e., an increase in indices value would reflect an increase in both thinning and thickening. Further, a careful examination of the EOR literature conveys that the Newtonian fluid, shear thinning, and shear thickening fluids would have a slope of 1, 0.1–1, and greater than 1, respectively [47]. The following section will compare the bulk rheological parameters with the porous media parameters. For these reasons, new shear thinning and thickening indices are calculated by adding 1 to those thinning and thickening indices shown in Figure 4 and Figure 7. The new shear thinning and shear thickening index for various concentrations are shown in Figure 8b. 

**Figure 9:**
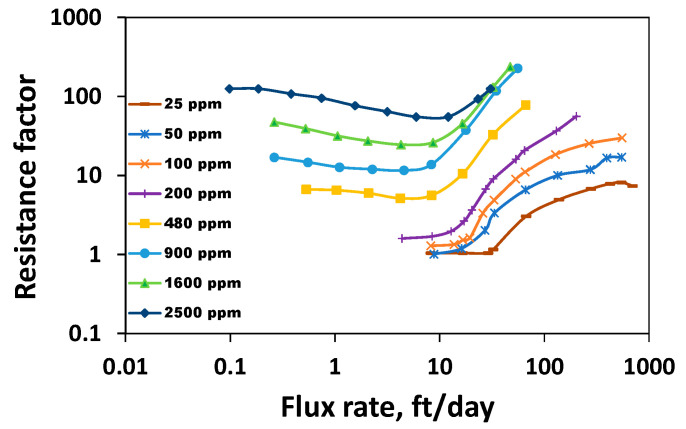
Porous media rheogram of HPAM 3830 systems at various concentrations from 25 ppm to 2500 ppm, adapted from [17,45].

While a moderate increase in Newtonian viscosity for the polymer systems having a lower HPAM concentration was noticed, from polymer concentrations above 200 ppm, the HPAM solutions exhibited a steep elevation of their viscosity values within the Newtonian regime (Figure 1). The shear characteristic time and shear thinning are zero for those concentration ranges belonging to the dilute regime. However, beyond the overlap concentration, the shear characteristic time increases (Figure 8a), and both the shear thinning and modified shear thinning index decrease (Figure 8b). While a decrease in the modified shear thinning index reflects an increase in the shear thinning intensity, an increase in characteristic time reflects an increase in the linear viscoelasticity. Unlike the shear thinning index in the dilute regime, which is zero, the shear thickening index shows an increase for an increase in concentration up to 100 ppm, but it is not shown in Figure 9. However, it is visible in Figure 1. This complies with the findings of Clasen [48], who reported that chains stretch in extensional flow within dilute regimes. Similarly, in high-speed shear flow, the polymer chain stretches even at concentrations within the dilute regime. Further, contrary to the increase in shear thinning after the overlap concentration, shear thickening begins to decrease. This is because entanglement at higher concentrations impairs the stretchability of the polymer systems. The viscoelastic onset rate remains relatively constant for concentrations higher than 200 ppm. Among all the parameters, only the shear thickening index shows a non-monotonic response; in other words, it shows an increasing–decreasing trend, and complies with the chain stretchability above and below CAC. The shear thickening index and porous media thickening index are compared in the next section. 

### 3.7. Relationship between Rheological Parameters in the Shear Field and Porous Media

To compare some rheological parameters concerning the same polymer systems flowing through porous media, on one hand, and under shear deformation, on the other hand, two data sets were used: one obtained from the results reported by Seright et al. (2011, HPAM solutions with concentrations ranging from 25 to 1600 ppm) [45] and Seright (2017, HPAM concentration of 2500 ppm) [17] and the other resulted from shear rheology carried out in this study (Figure 9). It is essential to highlight that the resistance factor is plotted on the y-axis of Figure 9. The resistance factor at a particular rate is defined as a measurement of a polymer’s resistance to flow in the specific porous media when compared to water’s resistance [55]. It is defined as

$$\mathrm{Resistance}\;\mathrm{factor}=\frac{{dP}_{polymer}}{{dp}_{water}}$$

where

${dP}_{polymer}$ is the stable pressure drop at a particular rate during polymer injection;
${dp}_{water}$ is the stable pressure drop at a particular rate during primary water injection.

The stable pressure drop polymer and water injection are obtained by ensuring the pressure drop remains constant for a specific pore volume of injection. Since the porous media and flow rate are the same, only viscosity differs for both fluids. If the water viscosity is 1 cP, and the effective permeability of the polymer is considered the same as the effective permeability of water, then the resistance factor is equivalent to the polymer’s viscosity. For the reservoir rocks whose permeability is above 200 mD, the residual resistance factor (indicative of the polymer’s retention) of the polymer is not significant [17]. Therefore, the effective permeability of water can be considered equivalent to the polymer’s effective permeability. This indicates that the resistance factor, despite being less, can be compared with the shear viscosity characterized by the unit of cP. The flux rate is equivalent to the velocity of the injection fluids in the porous media. The Newtonian viscosity, shear thinning index, viscoelastic onset rate, and shear thickening index are obtained from the provided in situ rheogram.

The porous media thickening index is extracted by fitting the power law to the shear thickening regime, as reported by Azad [37]. The porous media thinning/thickening index values were obtained by power-law-fitting the data from the shear thinning/thickening regime reported by Seright et al. [45] and Seright [17]. Once the fitting is complete, 1 is added to obtain the porous media thinning/thickening index. It is worthwhile to highlight that some viscoelastic models can predict the shear thickening behavior of EOR polymer solutions. 

**Figure 10:**
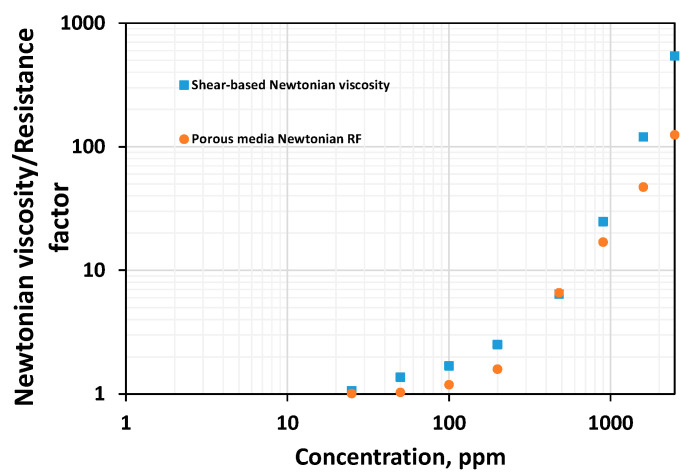
Comparison between the values of the Newtonian viscosity in shear and porous media for HPAM systems of various concentrations.

**Figure 11:**
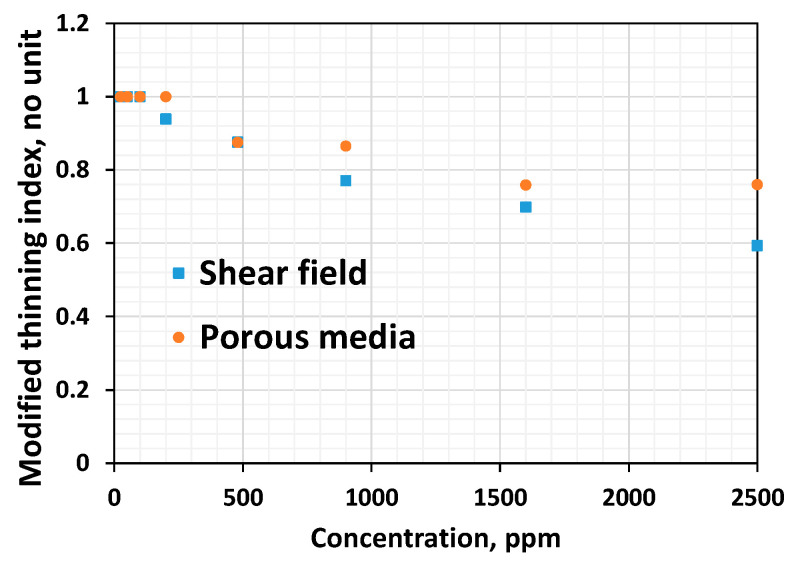
Comparison between the thinning indices in shear and porous media for HPAM systems of different concentrations.

**Figure 12:**
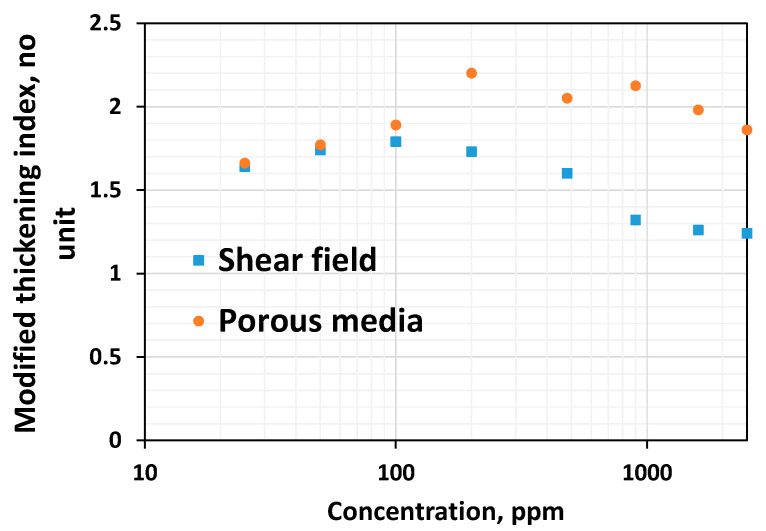
Comparison between the shear thickening index and porous media thickening index for HPAM systems of different concentrations.

**Figure 13:**
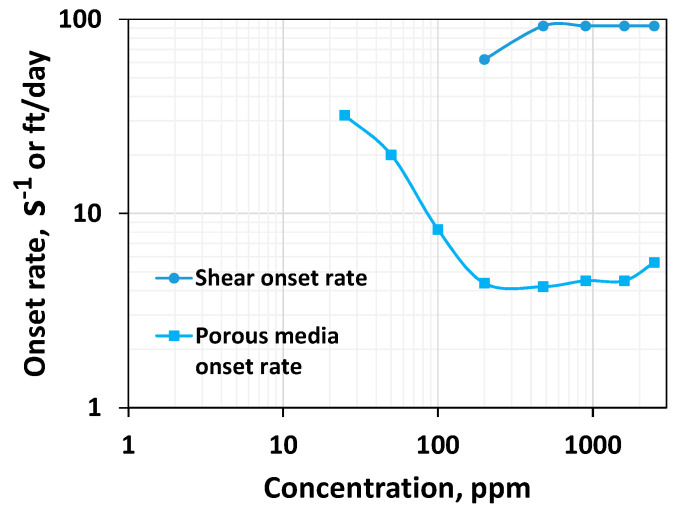
Comparison between the shear onset rate in the shear field and porous media for HPAM systems of different concentrations.

Figure 10 compares the Newtonian viscosity in both fields. Figure 11 compares the thinning index obtained by fitting the power law. Figure 12 and Figure 13 compare viscoelastic parameters such as the thickening index and onset rate in the shear field and porous media. Porous media thickening properties are extracted from the single-phase study performed using a similar HPAM system (HPAM 3830) by Seright et al. [45] and Seright [17]. The viscoelastic onset rate in the porous media is considered to be the rate at which shear thinning ends or shear thickening begins to occur.

Viscous properties such as the Newtonian viscosity are higher in the shear field when compared to the Newtonian resistance factor in porous media (Figure 10). The higher the shear thinning index, the lesser the thinning ability, and it appears that polymer solutions show stronger thinning in the shear field than in porous media (Figure 11). Viscous properties are those properties extracted from the Newtonian regime. Linear viscoelastic properties are those non-Newtonian properties extracted from low shear ranges. This includes the shear characteristic time. Non-linear viscoelastic properties are those extracted from the high-shear ranges. This includes the viscoelastic onset rate, shear thickening index, etc. 

While viscous properties manifest strongly in the shear field, non-linear viscoelastic properties manifest strongly in porous media. The lower the viscoelastic onset rate, the higher the non-linear elastic effects [37,47]. However, the higher the thickening index, the higher the viscoelasticity [37]. One common finding is that viscoelastic effects manifest with higher magnitudes in porous media compared to the bulk shear field (Figure 12 and Figure 13). This is because porous media has a strong extensional flow component that gives rise to stronger deformation due to the converging–diverging flow (size) effect and a subsequent stretch [37]. 

In both shear rheometry and porous media, the shear thickening index initially shows an increasing concentration of 100–200 ppm (Figure 12). A careful look at Figure 12 indicates that the thickening index at 100 ppm is slightly higher than the thickening index at 200 ppm. Nevertheless, after 200 ppm (CAC), the thickening index generally decreases in both fields, signifying that non-linear viscoelastic effects decrease concerning the increase in concentration above the CAC. This is consistent with the findings of Al-Hamad et al. [51], who employed special non-circular steady-shear rheometry to study the non-linear viscoelastic effects for 650 to 2350 ppm HPAM 6030. An increase in the viscoelastic onset rate, indicative of the decrease in non-linear viscoelastic effects for the increase in concentration, has been reported [51,56]. 

The viscoelastic onset rate in both the shear field and porous media remains constant at concentrations higher than a CAC of 200 ppm (Figure 13). One may wonder why at 2500 ppm the porous media viscoelastic onset rate is slightly higher. However, a careful look into Figure 13 of Seright [17] indicates that the resistance factors for 2500 ppm alone were measured at different rates, and therefore, a slight variation is seen at 2500 ppm. This suggests at concentrations higher than the CAC, the onset rate predicted by the shear rheometer qualitatively agrees with the porous media. What about the cases associated with lower concentrations? Although at concentrations less than 200 ppm, the viscoelastic onset in the shear field appears constant (Figure 1), an early onset is visible at lower concentrations in shear rheometry, which contradicts the porous media onset rate trend, which increases with respect to concentrations in the dilute regime (Figure 13). It suggests that the inertial effect might influence the onset rate at a concentration less than the CAC in shear rheometry, and this effect becomes weaker at a concentration higher than the CAC. This results in the onset rate in the shear field and porous media having the same trend in the shear field and porous media at higher concentrations as the constant onset rate trend. Although HPAM is the standard polymer used for EOR applications, other polymers, such as associative polymer and ATBS-based HPAM polymer, were evaluated [22]. As rheology is a complex phenomenon, it is imperative that both shear and porous media experiments be conducted with other polymers in the future to elucidate the influence of the CAC on their flow behavior.

Another interesting behavior observed is that low-concentrated polymer solutions (below the CAC) fail to show thinning in the shear field and porous media. In contrast, they show thickening both in the shear and porous media (Figure 1 and Figure 10). This suggests these dilute solutions lack linear viscoelastic effects, as evidenced by the zero characteristic time (Table 1) and lack of shear thinning (Figure 11), and possess non-linear viscoelastic characteristics both in the shear field and in porous media (Figure 12). 

## 4. Conclusions

The HPAM polymer system at various concentrations ranging from 25 ppm to 2500 ppm is characterized by its viscous and viscoelastic (linear and non-linear) properties in the steady-shear field and porous media. The following are the summarized conclusions.

1. Although the Newtonian viscosity increases monotonically with the increase in concentration in both the shear field and porous media, the degree of increase is higher above the critical overlap concentration.
2. Shear thinning and linear viscoelastic effects are absent for dilute solutions in both the shear field and porous media. However, thinning and linear viscoelastic effects increase at concentrations higher than the CAC, consistent with Howe et al. (2015).
3. At concentrations higher than 200 ppm, the onset rate remains almost constant in both the shear field and porous media. At lesser concentrations, the onset-rate behavior differs in porous media and bulk shear fields. This could be due to high inertia, and it also suggests that shear rheometry can be used to predict the polymer’s viscoelastic onset behavior effectively only if the concentration is higher than the CAC.
4. Up to the CAC, the shear and porous media thickening indices are similar and show an increasing trend at lower concentrations. However, immediately after 200 ppm, the shear thickening index decreases, whereas the porous media thickening index decreases after 980 ppm. Nevertheless, a further increase in concentration leads to a continual reduction in the porous media thickening index, consistent with the shear thickening index.

## Figures and Tables

**Table 1:** Rheological parameters of HPAM 3630 at various concentrations.

Polymer Concentration, ppm	Zero-Shear Viscosity (cP)	Shear Characteristic Time (s)	Modified Shear Thinning “Index,” (No Unit)	Onset of Viscoelastic Effects, (s^−1^)	Modified Shear Thickening “Index,” (No Unit)
25	1.06	0	0	N.A.	1.64
50	1.37	0	0	N.A.	1.74
100	1.69	0	0	N.A.	1.79
200	2.51	0.174	0.939	62.1	1.73
480	6.42	0.85	0.876	92.4	1.6
900	24.8	2.8	0.771	92.4	1.32
1600	120	30.39	0.699	92.4	1.26
2500	542	45.24	0.594	92.4	1.24

N.A.: For dilute concentrations, the onset rate varies slightly and appears to be influenced by inertial effects.

**Table 2:** Raw shear rheological data for HPAM 3630 at various concentrations.

Shear Rate, s^−1^	Shear Viscosity for 25 ppm, cP	Shear Viscosity for 50 ppm, cP	Shear Viscosity for 100 ppm, cP	Shear Viscosity for 200 ppm, cP	Shear Viscosity for 480 ppm, cP	Shear Viscosity for 900 ppm, cP	Shear Viscosity for 1600 ppm, cP	Shear Viscosity for 2500 ppm, cP
0.01	−10.661	−42.957	3.3782	14.973	32.455	−1.1092	149.07	485.25
0.0149	4.3064	−23.063	−5.8485	12.981	8.6885	45.658	135.13	542.13
0.0221	−2.2488	−13.142	−9.5952	16.508	10.514	32.135	120.18	555.1
0.0329	−1.3635	−12.751	−8.7618	3.5042	6.4289	24.8	123.62	534.7
0.0489	3.6576	−7.5204	−8.3424	3.2142	6.4848	25.582	118.88	506.9
0.0728	6.4044	−6.1574	−2.1003	1.107	7.5543	28.657	113.97	465.79
0.108	5.5594	−1.6433	−1.1504	2.5934	6.7435	24.638	108.68	424.78
0.161	5.1439	1.1499	1.0552	2.0768	5.8526	24.26	101.88	375.26
0.24	2.465	3.272	3.0898	2.0547	5.4804	23.282	94.432	326.41
0.356	−0.30535	2.2069	3.0226	2.808	5.8602	23.477	86.877	278.87
0.53	1.5398	0.58254	0.92371	2.661	5.9351	22.714	78.663	236.22
0.788	0.71415	1.8445	2.2393	2.5752	5.7039	21.236	69.837	198.88
1.17	1.2182	1.6043	1.7973	2.5183	5.5753	19.761	61.456	165.46
1.74	0.97374	1.3772	1.6974	2.5582	5.5371	18.303	53.42	135.88
2.59	1.0603	1.3068	1.5823	2.4931	5.3816	16.731	46.026	111.43
3.86	1.0239	1.2907	1.5784	2.4601	5.1731	15.14	39.363	91.359
5.74	1.0546	1.2727	1.5343	2.4145	4.9362	13.596	33.509	74.59
8.53	1.0433	1.263	1.5131	2.3464	4.6741	12.132	28.477	61.816
12.7	1.0511	1.2607	1.494	2.2775	4.407	10.779	24.273	51.747
18.9	1.0584	1.2583	1.4805	2.2083	4.1441	9.5686	20.994	43.408
28.1	1.0715	1.2631	1.4738	2.1518	3.897	8.544	18.367	37.662
41.8	1.0889	1.2725	1.4727	2.1136	3.686	7.9274	16.472	33.995
62.1	1.1152	1.2947	1.4825	2.1013	3.521	7.8179	15.35	30.221
92.4	1.1549	1.3401	1.5106	2.1339	3.416	7.7739	15.034	29.095
137	1.4184	2.1725	1.5742	2.3062	3.749	14.163	23.025	38.81
204	2.089	3.405	2.6225	5.5602	7.6178	15.715	25.685	39.57
304	2.9994	4.5839	4.0126	7.1173	9.3356	16.668	25.847	39.843
452	3.6625	5.7463	5.4009	8.2658	10.318	17.857	26.669	39.2
672	4.2641	6.8927	6.855	9.3356	11.71	18.68	28.29	38.099
1000	4.9696	8.1421	8.2629	10.859	13.434	19.99	28.269	36.673

## Data Availability

Data are contained within the article.

## References

[1] Hyne N. (2005). Dictionary of Petroleum Exploration, Production and Drilling.

[2] Taber J.J., Martin F.D., Seright R.S. (1997). EOR Screening Criteria Revisited—Part 1: Introduction to Screening Criteria and Enhanced Recovery Field Projects. SPE Res. Eng..

[3] Taber J.J., Martin F.D., Seright R.S. (1997). EOR Screening Criteria Revisited—Part 2: Applications and Impacts of Oil Prices. SPE Res. Eng..

[4] Towler B.F. (2002). Fundamental Principles of Reservoir Engineering.

[5] Terry R.E., Rogers J.B. (2014). Applied Petroleum Reservoir Engineering.

[6] Lee J., Rollins J.B., Spivey J.P. (2003). Pressure Transient Testing.

[7] Azad M.S. (2021). IFT Role on Oil Recovery during Surfactant-Based EOR Methods.

[8] Green D.W., Willhite P.G. (2017). Enhanced Oil Recovery.

[9] Delamaide E., Soe Let K.M., Bhoendie S., Pin J.A., Paidin W.R. Results of Polymer Flooding Pilot in the Tambaredjo Heavy Oil Field, Suriname. Proceedings of the SPE Canadian Heavy Oil Conference.

[10] Koning E.J.L., Mentzer E., Heemskerk J. Evaluation of a Pilot Polymer Flood in the Marmul Field, Oman. Proceedings of the 63rd Annual Technical Conference and Exhibition.

[11] Wang D., Wang G., Xia H. Large Scale High Visco-elastic Fluid Flooding in the Field Achieves Higher Recoveries. Proceedings of the SPE Enhanced Oil Recovery Conference.

[12] Delamaide E. Pelican Lake: Learning from the Largest Polymer Flood Expansion in a Heavy Oil Field. Proceedings of the Abu Dhabi International Petroleum Exhibition and Conference.

[13] Manichand R.N., Let M.S., Gil L., Quillien B., Seright R.S. Effective Propagation of HPAM solutions Through the Tambaredjo Reservoir During a Polymer Flood. Proceedings of the SPE International Symposium on Oil Field Chemistry.

[14] Seright R.S. (2017). How much polymer should be injected during polymer flood? Review of previous and current practices. SPE J..

[15] Zhao Y., Yin S., Seright R.S., Ning S., Zhang Y., Bai B. (2021). Enhancing Heavy-oil-recovery efficiency by combining low salinity water and polymer flooding. SPE J..

[16] Sagyndikov M., Seright R.S., Kudabergenov S., Ogay E. (2022). Field Demonstration of the impact of fractures on HPAM injectivity, propagation and degradation. SPE J..

[17] Seright R.S., Wang D. (2023). Polymer flooding: Current Status and Future Directions. Pet. Sci..

[18] Sagyndikov M.S., Kushekov R.M., Seright R.S. (2022). Review of Important Aspects and Performances of Polymer Flooding versus ASP Flooding. Bull. Univ. Karaganda..

[19] Asghari K., Nakutnyy P. Experimental Results of Polymer Flooding of Heavy Oil Reservoirs. Proceedings of the Canadian International Petroleum Conference.

[20] Cannella W.J., Huh C., Seright R.S. Prediction of xanthan gum rheology in porous media. Proceedings of the SPE Annual Technical Conference and Exhibition.

[21] Clarke A., Howe A.M., Mitchell J., Staniland J., Hawkes L.A. (2016). How Viscoelastic Polymer Flooding Enhances Displacement Efficiency?. SPE J..

[22] Taylor K.C., Nasreldin H.A. (1998). Water-soluble Hydrophobically Associating Polymers for Improved Oil Recovery: A Literature Review. JPSE.

[23] Azad M.S., Sultan A. Extending the Applicability of chemical EOR in high-salinity, high-temperature and fractured Carbonate reservoirs through viscoelastic surfactants. Proceedings of the SPE Saudi Arabia Section Technical Symposium and Exhibition.

[24] Levitt D.B., Pope G.A. Selection and Screening of Polymers for Enhanced oil recovery. Proceedings of the SPE Improved Oil Recovery Symposium.

[25] Barnes H.A., Hutton J.F., Walters K. (2010). An Introduction to Rheology.

[26] Hirasaki G.J., Pope G.A. (1974). Analysis of Factors Influencing Mobility and Adsorption in the Flow of Polymer Solution Through Porous Media. SPE J..

[27] Masuda Y., Tang K.C., Miyazawa M., Tanaka S. (1992). 1D Simulation of Polymer Flooding Including the Viscoelastic Effects of Polymer Solutions. SPE Res. Eng..

[28] Magbagbeola O.A. (2008). Quantification of the Viscoelastic Behavior of High Mw Polymers Used for Chemical Enhanced Oil Recovery. Master’s Thesis.

[29] Huh C., Pope G.A. Residual oil Saturation from Polymer Floods: Laboratory Measurements and Theoretical Interpretation. Proceedings of the SPE Improved Oil Recovery Symposium.

[30] Ehrenfred D. (2013). Impact of Viscoelastic Polymer Flooding on Residual Oil Saturation in Sandstones. Master’s Thesis.

[31] Qi P., Ehrenfred D.H., Koh H., Balhoff M.T. (2017). Reduction of Residual Oil Saturation in Sandstone Cores by Use of Viscoelastic Polymers. SPE J..

[32] Erinick M.Z., Qi P., Balhoff M.T., Pope G.A. (2018). New Method to Reduce Residual Oil Saturation by Polymer Flooding. SPE J..

[33] Hincapie R.E., Rock A., Wegner J., Ganzer L. Oil Mobilization by Viscoelastic Flow Instabilities Effects during Polymer EOR: A Pore-Scale Visualization Approach. Proceedings of the SPE Latin America and Caribbean Petroleum Engineering Conference.

[34] Azad M.S., Trivedi J.J. (2020). Extensional Effects During Viscoelastic Polymer Flooding: Understanding the Unresolved Challenges. SPE J..

[35] Azad M.S., Trivedi J.J. (2021). Quantification of Sor Reduction during Polymer Flooding using Extensional Capillary Number. SPE J..

[36] Azad M.S., Trivedi J.J. (2019). Quantification of Viscoelastic Effects during Polymer Flooding: A Critical Review. SPE J..

[37] Azad M.S. (2023). Characterization of Non-linear Viscoelastic Properties of EOR polymer systems using Steady-Shear Rheometry. SPE J..

[38] Ferguson J., Walter K., Wolff C. (1990). Shear and Extensional flow of Polyacrylamide Solutions. Rheol. Acta.

[39] Delshad M., Kim D.H., Magbagbeolo O.A., Huh C., Pope G.A., Tarahhom F. Mechanistic Interpretation and Utilization of Viscoelastic Behavior of Polymer Solutions for Improved Polymer-Flood Efficiency. Proceedings of the SPE Improved Oil Recovery Efficiency Symposium.

[40] Zamani N., Bondino I., Kaufmann R., Skuage A. (2015). Effect of Porous Media Properties on the Onset of Polymer Extensional Viscosity. JPSE.

[41] Howe A.M., Clarke A., Giernalczyk D. (2015). Flow of Concentrated Viscoelastic Polymer Solutions in Porous Media: Effect of Mw and Concentration on the Elastic Turbulence Onset in Various Geometries. Soft Matter.

[42] Doshi S.R., Dealy J.M. (1987). Exponential Shear: A Strong Flow. J. Rheol..

[43] Wagner M.H., Garrido V.H., Chai C.K. (2015). Exponential shear flow of branched polyethylenes in rotational parallel plate geometry. Rheol. Acta.

[44] Jouenne S., Heurteux G. (2020). Correlation of Mobility Reduction of HPAM Solutions at High Velocity in Porous medium with Ex-situ Measurements of Elasticity. SPE J..

[45] Seright R.S., Fan T., Wavrik K., Balaban R.D.C. (2011). New Insights into Polymer Rheology in Porous Media. SPE J..

[46] Vermolen E.C.M., Almada M.P., Wassing B.M., Ligthelm D.J., Masalmeh S.K. Low-salinity polymer flooding: Improving polymer flooding technical feasibility and economics by using low-salinity make-up brine. Proceedings of the International Petroleum Technology Conference, IPTC 17342.

[47] Heemskerk J., Rosmalen R., Jannseen-Van R., Teeuw D. Quantification of Viscoelastic Effects of Polyacrylamide Solutions. Presented at the SPE Enhanced Oil Recovery Symposium, SPE 12652-MS.

[48] Clasen C., Plog J.P., Kullicke W.M., Owens M., Macosko C., Scriven L.E., Verani M., Mckinley G.H. (2006). How dilute are dilute solutions in extensional flow?. J. Rheol..

[49] Jouenne S., Levache B. (2020). Universal Viscosifying Behavior of Acrylamide-based Polymers used in Enhanced Oil Recovery. J. Rheol..

[50] Larson R.G., Shaqfeh E.S.G., Muller S.J. (1990). A Purely Elastic Instability in Taylor-Couette Flow. J. Fluid. Mech..

[51] Al-Hamad J., Azad M.S., Farhan M., Al-Shehri D., Barri A. (2023). Does Non-Circular Shear Rheometry Amplifies the Non-linear viscoelastic effects for an Improved Polymer EOR selection criteria?. Arab. J. Sci. Eng..

[52] Vermolen E.C.M., Haasterecht M.J.T., Masalmeh S.K. A Systematic Study of the Polymer Viscoelastic Effect on Residual Oil Saturation by Core Flooding. Proceedings of the SPE EOR Conference.

[53] Seright R.S., Seheult M., Talashek T. (2009). Injectivity Characteristics of EOR Polymers. SPE Reserv. Eval. Eng..

[54] Graessley W.W. (1974). The entanglement concept in polymer rheology. Adv. Polym. Sci..

[55] Pye D.J. (1964). Improved Secondary Recovery by Control of Water Mobility. J. Pet. Technol..

[56] Lewandowska K. (2007). Comparative Studies of Rheological Properties of Polyacrylamide and Partially hydrolyzed polyacrylamide Solutions. J. Appl. Polym. Sci..

